# The development of a health-related quality-of-life instrument for young people with narcolepsy: NARQoL-21

**DOI:** 10.1186/s12955-017-0707-8

**Published:** 2017-07-04

**Authors:** John E. Chaplin, Attila Szakács, Tove Hallböök, Niklas Darin

**Affiliations:** 1 Department of Pediatrics, Institute of Clinical Sciences, Sahlgrenska Academy at University of Gothenburg, Gothenburg, Sweden; 2 Department of Pediatrics, Halland Hospital, Halmstad, Sweden

**Keywords:** Narcolepsy, Child, Adolescence, HrQoL, Focus groups, Factor analysis, Roc, Mixed methods

## Abstract

**Background:**

Narcolepsy is a lifelong sleep disorder with a prevalence of between 0.03% and 0.06% and onset at around puberty. It is associated with psychiatric comorbidities and cognitive difficulties. No valid and reliable condition-specific health-related quality-of-life (HrQoL) instrument has been developed for this population.

**Methods:**

A questionnaire based on four mixed-gender age-defined focus group discussions and a patient panel analysis was administered to young people with narcolepsy and a control group. External reliability was measured by a test-retest procedure and internal reliability was measured using Cronbach’s alpha. Convergent validity with the KIDSCREEN-10 index was assessed using with intraclass correlation coefficients (ICC) and receiver operating characteristic (ROC) curves. Factor analysis techniques were used to identify suitable items and confirm the factor structure. Baseline values were assessed for convergent validity, ceiling effects, agreement and sensitivity. Comparison with KIDSCREEN-10 was made on the basis of area under the curve (AUC).

**Results:**

One hundred young people with narcolepsy and 95 control subjects returned questionnaires. The factor structure revealed two main factors with five domains and 21 questions, which was confirmed with confirmatory factor analysis. The domains of the NARQoL-21 showed good independence while the floor and ceiling effects were acceptable. The external reliability (0.928), convergent validity (rs = 0.769) and internal consistency (Cronbach’s alpha = 0.886) were excellent. A Bland–Altman plot revealed some proportional bias. Good discriminant validity was detected for control/patient (Cohen’s d = 2.114). ROC analysis showed significantly better AUC for NARQoL-21 (0.939) than KIDSCREEN (0.877). A cut-off score equivalent to KIDSCREEN-10 for suboptimal HrQoL which maximized sensitivity (84%) and specificity (92%) was found at NARQoL-21 score below 42.

**Conclusions:**

Establishing the validity of a disease-specific HrQoL instrument in a population of people with a rare condition poses significant challenges. The mixed-methods approach adopted here has resulted in a questionnaire of 21 items with good discrimination and convergent validity, and excellent internal and external reliability, allowing precise and stable measurements. The cut-off score can be useful to identify patients with very poor HrQoL and thus improve the design of treatment options. Further testing in a longitudinal cohort is recommended in order to establish responsiveness.

**Electronic supplementary material:**

The online version of this article (doi:10.1186/s12955-017-0707-8) contains supplementary material, which is available to authorized users.

## Background

Narcolepsy is a rare chronic neurological condition characterized by daytime sleepiness with repeated lapses into sleep of short duration [1]. The symptoms can include cataplexy (sudden episodes of muscle weakness triggered by strong emotions), sleep paralysis, and hypnagogic or hypnopompic hallucinations [2]. Associated features such as automatic behavior [3] may also be present and disrupted nocturnal sleep occurs in more than 50% of cases [1].

Narcolepsy has a prevalence ranging between 0.03% and 0.067% [4, 5] and a bimodal onset pattern with a large peak around puberty and a smaller peak around 35 to 45 years of age [6]. The disease is caused by deficiency of the sleep–wake regulating neuropeptide hypocretin-1 [7]. Narcolepsy has been associated with an increased prevalence of psychiatric comorbidity as well as cognitive difficulties [8, 9], which can lead to poor school performance [10, 11]. Adult patients report substantially lower health-related quality of life (HrQoL) indicating potential long-term consequences [12, 13]. Impaired HrQoL has also been found in studies of children with narcolepsy [10, 14]. Condition-specific measures are more sensitive for the detection and quantification of small changes that are important to patients and clinicians [15]; however, no condition-specific instruments have been developed to measure HrQoL in children or adolescents with narcolepsy. Therefore, in order to assess the particular concerns of this group, a self-report disease-specific instrument was considered important.

The purpose of this study is to describe the development of a narcolepsy-specific HrQoL self-report questionnaire for children and adolescents from conceptual model development through instrument validation. In order to incorporate the patients’ perspectives on the HrQoL outcomes that matter to them, a cooperative design process was used [16], which involved patient input via focus groups as well as patient participation in the design process via a patient panel.

## Methods

### Focus groups and item extraction

Four, age-defined (8–13 and 15–17 years), mixed-gender focus group interviews were carried out at the Queen Silvia Children’s Hospital, Gothenburg, and at the Ågrenska, National Competence Center For Rare Diseases, Gothenburg during 2012–2013. All participants spoke Swedish as their first or second language. A standardized focus group methodology developed for the DISABKIDS QoL project was used [17], including a semi-structured interview schedule using a multi-stage method converging on specific QoL issues related to narcolepsy [18]. The four sessions were held over approximately 90 min. All focus group discussions were digitally recorded and transcribed. Two additional focus groups were conducted with parents of these patients. A patient panel provided a thematic analysis of the statements, producing a set of themes which helped to identify important statements and reduce the number of items. Cognitive debriefing of the items further refined the statements. (See Focus Group Summary in Additional file 1.)

### Pilot study

The pilot questionnaire was distributed to patients and the control group during 2013. The patient participants were approached via the Swedish Narcolepsy Association (Narkolepsiföreningen), via hospital outpatient clinics in the health care regions covering the west of Sweden, and via a narcolepsy closed-group Facebook page. The control group was drawn from pupils and students attending local schools.

### Instruments used in the study

The KIDSCREEN-10 Mental Health Index was developed by means of a Rasch analysis, which ensured that only those items which represented a global, unidimensional latent trait were included. Rasch person parameters are assigned to each possible sum score, and transformed into values with a mean of 50 and standard deviation of 10. The index provides good discriminatory power and strong internal consistency (Cronbach’s alpha = .82) and test-retest reliability (*r* = .73), allowing precise and stable measurements [19, 20]. The cut-off at T-value below 38 (which represents the lowest 10%) indicates higher risk for poor mental health [19]). All analyses are done with 10-item General HRQoL index international T-values based on RASCH person parameters. A Swedish language version of the index was developed and validated as part of the original KIDSCREEN project [19].

The NARQoL-21 is a HrQoL instrument for children and adolescents with narcolepsy, consisting of 21 questions in five domains. These are summarized into the three following measures: Psychosocial summary scale, Future outlook summary scale, and HrQoL scale. It is a child self-report instrument using a five-point Likert scale with responses ranging from completely untrue to completely true. The summated rating method is used for each domain and, for ease of interpretation, the raw scores are transformed to a 0–100 scale, with higher scores indicating a positive HrQoL.

### Translation

An English translation of the NARQoL-21 (See Additional file 2) was carried out through several steps: an initial forward translation (from Swedish to English) by two independent translators with a background in linguistics and quality of life research, a back translation (from English to Swedish), and finally a consensual version to check against the original NARQoL-21 by all three translators and the originator of the instrument. Discrepancies were discussed and agreement achieved.

### Psychometric testing

Data were analyzed using IBM Corp. Released 2011. IBM SPSS Statistics for Windows, Version 20.0. Armonk, NY: IBM Corp. To test the external reliability of the questionnaire, a test–retest procedure was carried out with an interval of 2 weeks between questionnaire completion. Respondents were contacted via the Swedish Narcolepsy Association members who had completed the questionnaire. There was no preselection of respondents for participation in this stage. Internal reliability was assessed by Cronbach’s alpha. A classical test theory approach was used to analyze the pilot test results and identify items for deletion. Floor and ceiling effects are considered to be present if more than 15% of respondents achieve the lowers or highest possible score [21]. Means and SD of scores for matched patient and control groups are presented in order to enable interpretability of scores.

Both principal components and principal axis factor analyses with Promax rotation were undertaken. Three criteria were used to determine the number of factors: scree plot, eigenvalue, and percentage of variance explained by the factors. A threshold factor loading of 0.5 was chosen as a good indicator of sample-to-population pattern fit. The best-fitting model identified from the exploratory factor analysis was subsequently submitted for confirmatory factor analysis using IBM SPSS AMOS for Windows version 20.0.0.1. The overall model fit was assessed using the chi-square test statistic and alternative fit indices with the following cut-offs for acceptable fit: below 0.08 for the root mean squared error of approximation (RMSEA), above 0.90 for both the comparative fit index and the Tucker–Lewis index, while factor loadings should be above 0.40 [22].

### Missing data

Complete case analysis (listwise deletion), was applied for analyses of the pilot test data. Cases were removed where missing data exceeded 10% and affected the analysis. The largest amount of missing data was due to the control group not completing both the NARQoL-21 and KIDSCREEN-10 questionnaires. Subjects not completing both questionnaires were excluded from control group comparisons thus resulting in different numbers at different points in the analysis.

### Reliability

Item analyses included inspecting item means, medians, standard deviations, percentage ceiling and floor effects, item-to-total correlations, and Cronbach’s alpha to determine internal consistency. A 2-week test-retest reliability test was posted to a subgroup of patients from the Swedish Narcolepsy Association. External reliability was assessed using an intraclass correlation coefficient (ICC) on a two-factor mixed-effects model [23] with 95% confidence interval (CI). An ICC of at least 0.70 was considered to be satisfactory for group comparisons, whereas an ICC of at least 0.90 was considered to be satisfactory for individual comparisons [24].

### Construct validity

Convergent validity was assessed by examining the association between KIDSCREEN-10 Mental Health Index and the new NARQoL-21 questionnaire at the scale level. In the comparison of generic and condition specific instruments and based on previous research, we hypothesized that the KIDSCREEN-10 would show moderate-to-strong positive correlation coefficients [19, 25]. We used the Spearman rank correlation (rs). However, we were interested in more than just correlation between the two measures and wished to measure the strength of the agreement between them [26]; therefore, we assessed the ICC and Bland–Altman plot [26]. The ICC was computed with a two-way random effects model based on absolute agreement and a coefficient above 0.7 as an indicator of strong agreement [27]. To visualize the agreement, we represented the data graphically in a Bland–Altman plot [26].

Known groups analysis was conducted by comparison between a patient group and a control group. These analyses detected significant differences in the median scores between the two groups (Mann–Whitney U test). The effect size, indicating the degree of differences between the groups, was calculated by dividing the Standardized Test statistic (z) by the square root of N (where N is the number of observations). According to Cohen [28], for independent samples, the degree of difference (d value) of 0.20 is small, 0.50 is medium, and 0.80 is a large. Cohen’s d was calculated with Becker Effect Size Calculator [29].

### Sensitivity of the NARQoL-21

A receiver operating characteristic (ROC) analysis was conducted to describe the sensitivity and specificity of the NARQoL-21 compared to the criteria measure of KIDSCREEN-10. The area under the ROC curves (AUC) was computed to compare the discriminative properties of the two instruments [30]. The ROC method provides a useful overview of the relationship between a measure and an external indicator of health [31]. Thereby, the validity of the new questionnaire will be reflected by its ability to discriminate between healthy control subjects and patients with narcolepsy. A z-score test was used to compare the AUCs, and a value of *p* < 0.05 (one-tailed test) was considered a significant difference [32].

## Results

### Content validity: Focus groups, patient panel, and cognitive debriefing

**Fig. 1:**
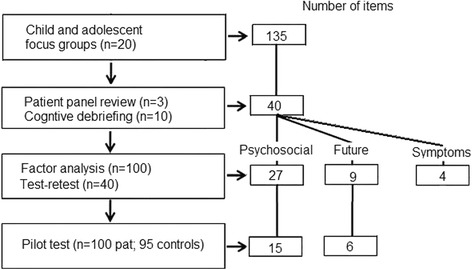
Item reduction procedure

Children and adolescents with narcolepsy identified in the health care regions covering the west of Sweden participated in four focus groups. In total, twenty young people with narcolepsy (age range 8–18; mean: 13.5 years) participated in age-defined (8–13 and 15–17 years) mixed gender focus groups with two additional focus groups for parents. Narcolepsy onset was between one and two years prior to participation. The initial analysis produced seven themes with 135 items related to the concept of their HrQoL and submitted for review and thematic analysis by the a patient panel consisting of three representatives of the Swedish Narcolepsy Association; as a result, themes were clarified, questionnaire items were rewritten to improve understanding, and the number of items was reduced. Another part of the review process was a cognitive debriefing with 10 adolescents with narcolepsy attending the Ågrenska center. The focus group methodology and analysis are presented in the focus group report (Additional file 1). At the end of the qualitative stage, a subset of 40 important and relevant items with good content validity were selected for inclusion in the pilot questionnaire. These 40 items comprised 27 HrQoL questions, nine questions relating to the future, and four questions related to symptoms (Fig. 1). The cognitive debriefing exercise indicated that the questions were acceptable and understood as expected, but minor rewording was suggested. Neither the cognitive debriefing participants nor the patient panel considered any of the questions to be too difficult for the targeted age range. The themes were: Emotional support, School performance, Social image, Concern about the future, Being limited by the condition, Personal energy, and Disturbed sleep.

### Pilot test

A total of 100 questionnaires were returned from patients with narcolepsy (59 girls) recruited from the Swedish Narcolepsy Association (*n* = 29), hospital outpatient clinics in the health care regions covering the west of Sweden (*n* = 38), and via a narcolepsy closed-group Facebook page (*n* = 33) (Table 1). The patients met the diagnostic criteria for narcolepsy according to ICSD-3 [33] . Narcolepsy type 1 was confirmed in 85% of the patients. In the other patients, cataplexy was absent or cerebrospinal fluid hypocretin had not been measured.

**Table 1:** Demographic data on 100 patients with narcolepsy

Gender (%)	Female	59
	Male	41
Age at onset (mean ﻿± SD)		13.2 ± 2.5
Age at diagnosis (mean ﻿±﻿ SD)		14.5 ± 3.2
Age at study (mean ± SD)		15.8 ± 4.3
Symtoms	ESS score (mean ± SD)	13.0 ± 4.4
	Cataplexy (%)	85
	Hallucination (%)^a^	52
	Sleep paralysis (%)	43
	Disturbed sleep (%)	86
Treatment	Methylphenidate (%)	87
	Modafinil (%)	21
	Amphetamine (%)	﻿7
	Antidepressants (%)	21
	Caffeine tablets (%)	7
	Sodium oxybate (%)	39

*Abbreviations: SD* standard deviation, *ESS* Epworth Sleepiness Scale

^a^hypnagogic or hypnopompic hallucinations

The items were assessed for normality. Three items had a skewness value of greater than the prior set criterion value of 2.0 and were therefore rejected, resulting in a total of 15 items for the Psychosocial summary scale and six items for the Future outlook summary scale. The item reduction procedure is presented in Fig. 1.

### Factor structure

Both principal components and principal axis factor analyses were conducted to discover the latent nature of the divisions in the HrQoL data. Three criteria were used to determine the number of factors: scree plot, eigenvalue, and percentage of variance explained by the factors. A minimum of four items loading higher than 0.5 within each factor was set as the criterion for a usable factor. A solution based on a fixed number of factors was tested with different rotation solutions. A three-factor solution (Emotional, School/Concentration, and Social) gave the best description of the Psychosocial items and a two-factor solution (Expectations and Limitations) gave the best description for the Future outlook items. See Table 2 for the Psychosocial factor structure and Table 3 for the Future outlook factor structure.

**Table 2:** Psychosocial factor structure

	Factor structure		
Item titles	Emotional reaction	School / Concentration	Social confidence
Sadness	0.771		
Feeling alone	0.717		
Anger	0.621		
Anxiety	0.602		
Irritability	0.537		
Ability to concentrate		0.708	
Staying awake when watching TV		0.664	
Alertness		0.615	
Alertness in school		0.588	
Ability to follow lessons		0.577	
Staying awake on the bus		0.545	
Feeling confident			0.607
Not giving up easily			0.573
Getting on with others			0.488
Feeling lazy			0.469

Extraction method: principal axis factoring

Rotation method: PROMAX with Kaiser normalization

**Table 3:** Future outlook factor structure

	Factor structure	
Item titles	Future expectations	Future limitations
Hopeful future	0.990	
Possibilities	0.611	
Problems in the future	0.465	
After leaving school		0.613
Travel		0.593
Driving license		0.539

Extraction method: principal axis factoring

Rotation method: PROMAX with Kaiser normalization

**Fig. 2:**
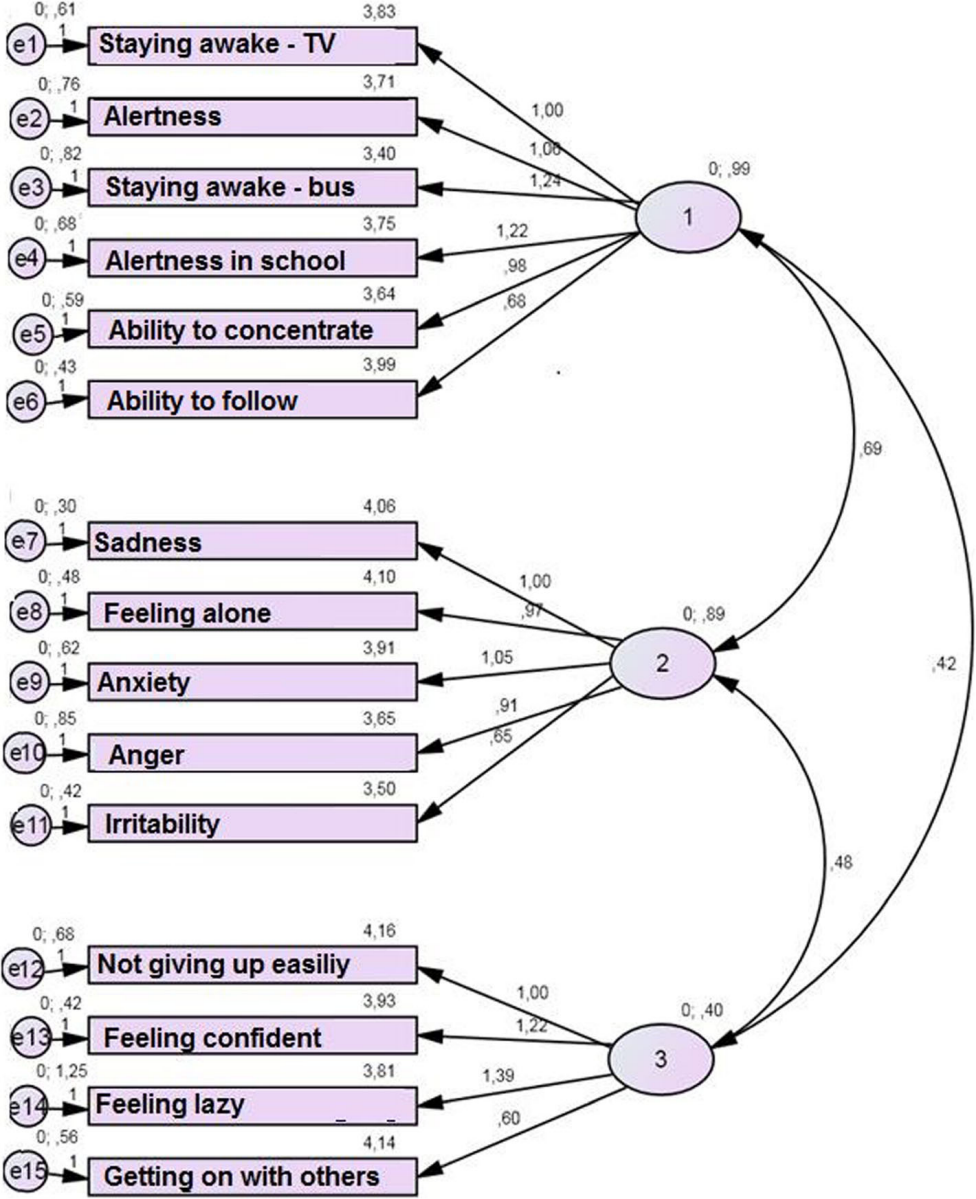
Confirmatory factor analysis path diagram (*n* = 195)

The resulting questionnaire consisted of 21 items in two factors (Psychosocial, Future outlook) with five domains (Emotion, Social, School/Concentration, Expectations, Limitations). The patient panel in the review, and the adolescents with narcolepsy in the cognitive debriefing, considered that a questionnaire of 21 questions would not pose a significant burden, even on younger patients. The questions used in NARQoL-21 can be found in Additional file 2 **.** A confirmatory factor analysis was conducted to assess the factor structure on the total population (*n* = 195). Maximum likelihood with estimated means and intercepts was used because of missing data. The chi square/degrees of freedom ratio was 2.03, which was less than the threshold of 3.0 (*χ* ^2^ = 176.4, df = 87, *p* < .001); the comparative fit index was 0.94 and the Tucker–Lewis index was 0.919, both greater than the cut-off minimum value of 0.90, thus indicating a reasonable-to-good model fit. The root mean square error of approximation was 0.073; and probability of close fit was 0.009, indicating that the overall model fit between the hypothesized model and the data was moderate to good. Therefore, item factor loadings were considered satisfactory. See Fig. 2 for the confirmatory factor analysis path diagram.

A correlation analysis showed independence of the three HrQoL domains. The highest correlation was between Emotional reaction and Expectations for the future (0.629) (see Table 4).

**Table 4:** Correlations between domains (*n* = 100 patients)

	Psychosocial domains			Future outlook domains	
	Emotional reaction	Social confidence	School/ Concentration	Expectations	Limitations
Emotional reaction	1	0.461	0.388	0.629	0.448
Social confidence		1	0.242	0.512	0.429
School/ Concentration			1	0.509	0.335
Expectations				1	0.515
Limitations					1

All correlations are significant at the 0.01 level

### Descriptive characteristics of the NARQoL-21 (*n* = 158)

The NARQoL-21 mean transformed score was 58 (SD 16), Psychosocial 56 (SD 15.8), and Future outlook 64 (SD 22). The floor and ceiling results were acceptable, although the Limitations domain stood out from the others, with a ceiling of 18%. The Cronbach’s alpha value (indicating internal reliability) was 0.886 for the NARQoL-21, 0.832 for Psychosocial, and 0.798 for Future outlook. The lowest Cronbach’s alpha value was found for the social domain (0.625) (Table 5). The response distribution was considered to be optimal.

**Table 5:** Characteristics of the NARQoL-21 scales in the study sample

	Patient group *n* = 79 (mean age 14 yrs)				Control group *n* = 79 (mean age 12 yrs)	Total sample *n* = 158
Scale (no. of items)	Mean (SD)	% floor	% ceiling	alpha	Mean (SD)	alpha
HrQoL (21)	58.2 (16.3)	0	0	0.886	88.1 (9.8)	0.938
Psychosocial (15)	55.9 (15.8)	0	0	0.839	86.8 (11.1)	0.916
Future outlook (6)	64.0 (21.8)	0	2	0.776	91.1 (9.0)	0.832
Emotional reaction (5)	58.3 (22.7)	0	0	0.826	85.3 (13.5)	0.861
Social confidence (4)	66.8 (19.7)	0	4	0.638	83.4 (16.0)	0.695
School/Concentration (6)	46.8 (20.1)	0	0	0.761	90.4 (9.8)	0.888
Expectations (3)	55.8 (25.0)	0	6	0.786	87.5 (13.6)	0.837
Limitations (3)	72.3 (24.9)	1	18	0.629	94.8 (10.0)	0.669

### Test-retest reliability (*n* = 39)

A test–retest procedure was carried out on 40 respondents. One respondent did not complete the retest and was therefore excluded from the analysis. Due to the method of administration, follow-up of non-responders was not possible. According to the Swedish Narcolepsy Association, no selection was made and no bias in responders was evident.

Questionnaires were returned within an average of 15 days. Three patients had missing data of less than 10%, and these scores were imputed based on their individual range of scores. Mean ± SD scores for the test and retest were 57.39 ± 13.68 and 61.11 ± 14.04, respectively (Table 6).

**Table 6:** Test-retest results with intraclass correlation coefficients (ICC)

*N* = 39	Mean (SD)	Min	Max	ICC (95%CI)
NARQoL-21_1 (test)	60 (14)	33	100	0.928 (0.858–0.963)
NARQoL-21_2 (retest)	62 (15)	29	100	
Emotion_1	61 (19)	20	90	0.833 (.776–.938)
Emotion_2	63 (20)	10	95	
Social_1	69 (19)	19	100	0.869 (.751–.931)
Social_2	72 (18)	25	100	
School_1	47 (18)	17	92	0.924 (.855–.960)
School_2	52 (18)	21	96	
Expectations_1	59 (25)	8	100	0.906 (.822–.951)
Expectations_2	59 (24)	8	100	
Limitations_1	74 (22)	17	100	0.926 (.859–.961)
Limitations_2	72 (24)	17	100	

The ICCs (average measures) were good: NARQoL-21 = 0.933 (95% CI 0.873–0.965). The agreement between the measures was good to excellent with a similar standard error of the means.

### Floor and ceiling effects

As shown in Table 5, there were generally no floor or ceiling effects. The exception is in the Limitation domain, where a ceiling effect of 18% was found. This indicates that caution must be taken when interpreting the scores of patients with a high score (lack of limitation) on this domain, due to reduced reliability at this end of the scale.

### Convergent validity - patients only (*n* = 92)

NARQoL-21 showed good convergent validity against the KIDSCREEN-10 index. A correlation of rs = 0.769 and ICC (average measures) of 0.706 (CI 0.555–0.806) were found, indicating good convergent validity. This means that the NARQoL-21 can be used as a satisfactory measure of HrQoL in this population.

**Fig. 3:**
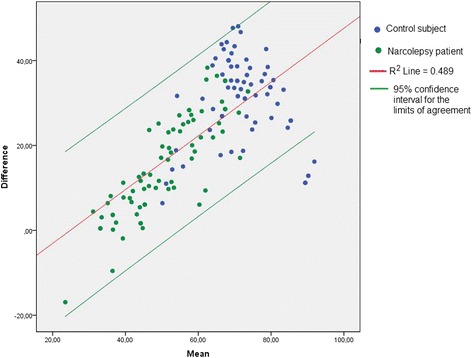
Bland–Altman plot showing differences between the NARQoL-21 and KIDSCREEN-10 tests

Comparing the patient and control populations, it was found that NARQoL-21 had a larger range of scores than KIDSCREEN-10 and a closer approximation to a normal distribution in the patient population (Table 7). A Bland–Altman plot (Fig. 3) shows that, despite the good correlation between the measures and the fact that 97.5% of the values were within the 95% confidence intervals (limits of agreement), there was proportional bias. This is shown by the scatter of difference values increasing progressively as the average values of the tests increase. This proportional bias can mostly be eliminated by log transformation of the raw values as recommended by Bland and Altman [34]; however, the bias was still not fully eliminated. As expected, the control subjects were mostly at the higher end of the HrQoL scale and it is here that the greatest variation between the tests was observed.

**Table 7:** Matched sample comparison of psychometric characteristics

Matched samples *n* = 79/79	Mean age; gender		Min–Max	Mean (SD)	Skewness	Kurtosis
Patient group	14 yrs.; 39 male 40 female	NARQoL-21	20–92	60 (16)	−0.108	−0.677
		KIDSCREEN-10	29–63	43 (7.2)	0.484	0.004
Control group	12 yrs.; 39 male 40 female	NARQoL-21	51–100	88 (10)	−1.603	2.975
		KIDSCREEN-10	38–84	56 (10)	1.180	1.165

### Discriminant validity

Discriminant validity was tested with known-group analysis on narcolepsy patients versus matched healthy controls, using a Mann–Whitney U test to determine whether the distribution of HrQoL in the two groups differed significantly (*U* = 405.5, *n*_1_ = *n*_2_ = 158, *P* < 0.001 two-tailed) with an effect size of d = 2.114 indicating a significant difference between the control and patient populations (Table 8).

**Table 8:** Discriminant validity using known groups (healthy controls vs narcolepsy patients)

Scale (no. of items)		Mean (SD)	Min–max	*P* value	Cohen’s d
HrQoL (21)	Patient	59 (10)	20–92		
	Control	88 (10)	51–100	<0.001	2.114
Psychosocial (15)	Patient	58 (16)	15–90		
	Control	87 (11)	42–100	<0.001	2.253
Future outlook (6)	Patient	65 (21)	13–100		
	Control	91 (9)	67–100	<0.001	1.641
Emotional reaction (5)	Patient	61 (23)	5–95		
	Control	85 (14)	25–100	<0.001	1.288
Social confidence (4)	Patient	67 (20)	13–100		
	Control	83 (16)	31–100	<0.001	0.892
School/Concentration (6)	Patient	49 (19)	17–88		
	Control	90 (10)	58–100	<0.001	2.774
Expectations (3)	Patient	57 (25)	8–100		
	Control	88 (14)	33–100	<0.001	1.503
Limitations (3)	Patient	73 (23)	17–100		
	Control	95 (10)	58–100	<0.001	1.243

### Sensitivity of the NARQoL-21

**Fig. 4:**
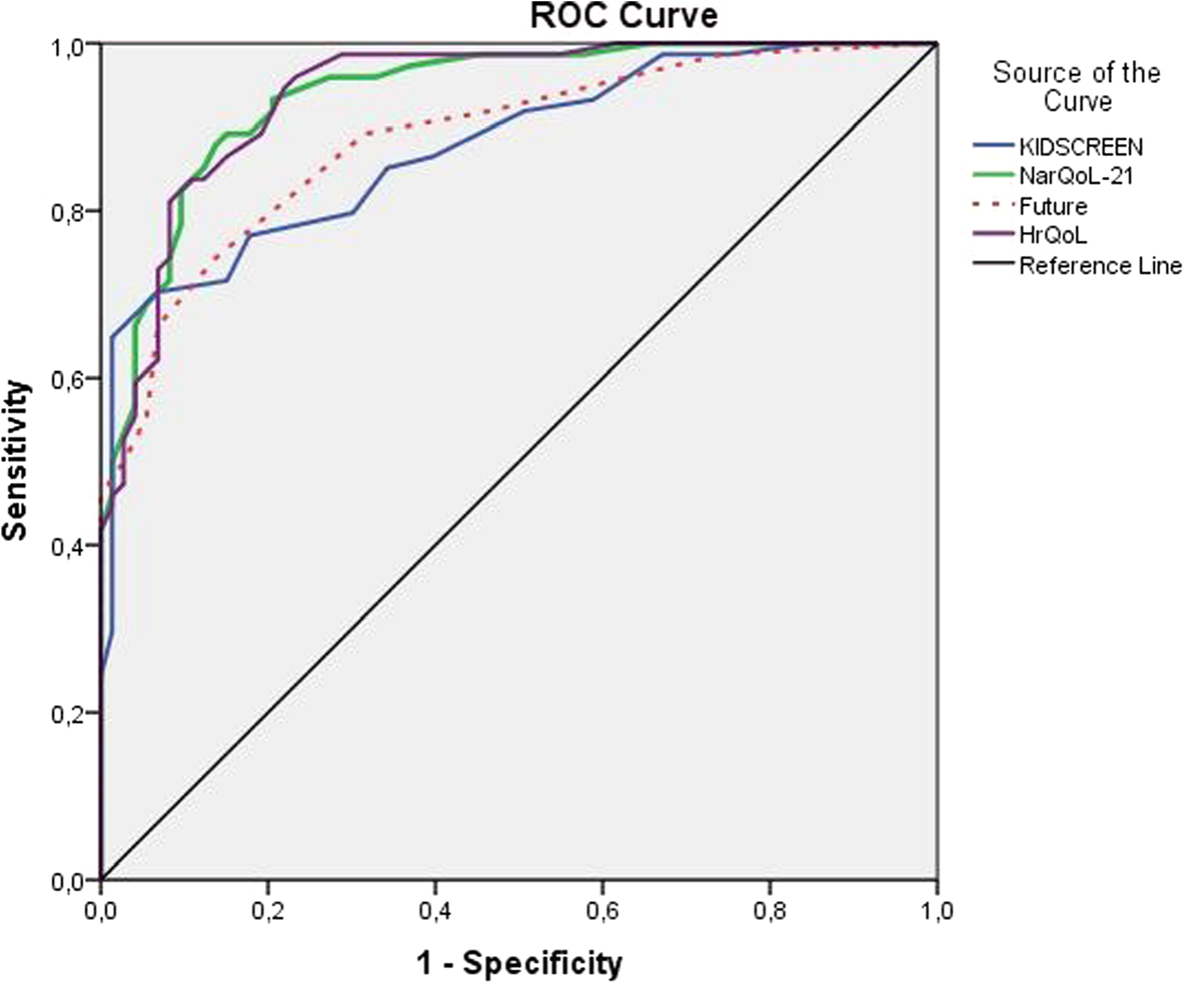
ROC curve comparing NARQoL-21 and KIDSCREEN-10 (*n* = 158)

A ROC curve was calculated to identify the relative sensitivity of the new HrQoL instrument (NARQoL-21) against the comparison instrument (KIDSCREEN-10). The AUC for the NARQoL-21 was 0.939 (CI 0.903–0.975), for the Psychosocial factor it was 0.940 (CI 0.904–0.976) and for the Future outlook factor 0.886 (CI 0.833–0.938). The AUC for KIDSCREEN-10 was 0.876 (CI 0.821–0.932) (Fig. 4).

Both instruments demonstrated excellent or good sensitivity and specificity; however, the NARQoL-21 had better discrimination of healthy controls. A z-score test showed a significant difference between the AUC for the two instruments (*p* = 0.038).

### Interpretability of scores – A clinical cut-off for intervention

The NARQoL-21 reached acceptable levels of reliability (>0.9) for individual assessment [24] and therefore the estimation of cut-off scores may be appropriate. Cut-off values for suboptimal HrQoL can also be identified via the ROC curve. Using the cut-off value of <38 provided by the developers of KIDSCREEN [19], adolescents were classified as having an optimal HrQoL score if it was above the cut-off point and a suboptimal score if it was below the cut-off point. In our population 19 had suboptimal HrQoL according to the KIDSCREEN-10 cut-off, all 19 were from the patient population. An equivalent cut-off score was selected that maximized the sum of sensitivity and specificity. A NARQoL-21 score of <42 would identify individuals with suboptimal HrQoL with a sensitivity of 84%, which corresponds to high negative predictive value, and specificity of 92%, which gives high specificity corresponding to high positive predictive value, thus having an acceptable likelihood of a false positive result (Table 9 in Additional file 3). At this cut-off, NARQoL-21 would be considered as having high sensitivity [35].

## Discussion

To our knowledge, the NARQoL-21 represents the first disease-specific HrQoL instrument for use in children and adolescents with narcolepsy. The NARQoL-21 is a brief questionnaire with two factors, five domains, and 21 items, which can be used as a tool to assess the patient-reported outcomes of narcolepsy treatment. The two factors are Psychosocial (15 items) and Future outlook (6 items). The NARQoL-21 is not limited by floor or ceiling effects, which means that the instrument does not limit the range of data that can be reported within this population. The item list was developed from the expressed views of children and adolescents with narcolepsy, aged 8–18 years. The final pilot questionnaire was refined on the basis of patient panel discussions with representatives of the Swedish Narcolepsy Association and on psychometric analysis. It is relatively short and has acceptable patient burden. A score of <42 was selected as a cut-off score that maximized the sum of sensitivity and specificity. The NARQoL-21 provides a good discriminatory power and it is a valid and well-tested, stable, child-centred, self-report measure for children and adolescents with narcolepsy.

The questionnaire differs from many instruments measuring HrQoL by the inclusion of two domains related to future expectations and limitations. The focus groups produced an image of the young people’s attitudes to their condition which went beyond the traditional model of HrQoL and included aspects of concern for the long-term future (see Additional file 1). These issues demonstrate a strong sense that these young people feel that they will miss out on what could be called a ‘good life’. This is not an entirely unique idea within HrQoL research, although concerns about the future are not normally included as domains in HrQoL measurement. The idea of including future outlook in an assessment of HrQoL has been explored by others [36, 37]. Raphael et al. [36] categorized HrQoL into three areas: *being* (the basic aspects of who the person is), *belonging* (the person’s fit with his or her environment), and *becoming* (the purposeful activities carried out to achieve personal goals, hopes, and wishes) [38, 39]. In our model, *being* and *belonging* come within the concept of the Psychosocial domains (Emotional, Social, and School/Concentration) whereas the *becoming* concept is captured by the two Future outlook domains (Expectations and Limitations). Future outlook is clearly an important consideration for children and adolescents.

Even patients with a relatively low burden of narcolepsy can experience a decrease in their HrQoL due to the occupational, career, and social effects of narcolepsy [40]. This highlights the need for a condition-specific measure that can identify outcomes from the perspective of the children themselves [41]. There have been very few HrQoL studies on children and adolescents with narcolepsy, and those that have been carried out have so far used generic instruments. For example: Stores et al. [14] used the parent version of the Child Health Questionnaire (age range 5–17 years; 50-items; 13 domains including two domains covering limitations in schoolwork and activities with friends [42]; Inocente et al. [43] and Lecendreux [44] used the self-report version of the Experience and Perceived Health in Adolescents instrument (Vécu et Santé Perçue de l’Adolescent, VSP-A, age range 11–17 years; 39 items; 7 domains, including school life and future) [37]; Rocco et al. [11] used the self-report version of the Pediatric Quality of Life Inventory (PedsQL 4.0, age range 5–18 years; 23 items, 4 domains: physical, social, emotional and school functioning) [45]. All of these instruments are generic HrQoL instruments. The PedsQL 4.0 has a domain structure similar to NARQoL-21, with physical, emotional, social and school functioning, and the VSP-A has a future domain similar to NARQoL-21, however neither of these instruments are condition specific. Results derived from the NARQoL-21 can be used to analyze which aspects of disease have the strongest effect on the patient. We would suggest that this will better facilitate the provision of personalized care and increase patient satisfaction.

Some limitations of the study should be recognized. Firstly, the study sample was relatively small, as is inevitable when working with rare-condition populations. A single study of this kind can only provide limited support towards establishing reliability or validity. Secondly, in relation to the challenge above, we prioritized inclusion in order to increase the number of participants in the study but, due to the practicalities of access to patient and control subjects, it was not possible to match the control group by age exactly to the patient group; however, by excluding the oldest 21 patients from the patient group, a substantial subset of the participants could be matched adequately for the analyses described here. There is still a difference in age, although we do not believe that this will affect the analyses carried out. Thirdly, the heterogeneity of the patient population, having been drawn from several different sources, and the fact that this is a cross-sectional study, will limit the inferences that can be drawn. Nonetheless, it can be deduced that there is substantial evidence of reasonable convergent validity with the generic instrument (KIDSCREEN-10) and that the NARQoL-21 could be used to assess young narcolepsy patients’ subjective QoL. The true responsiveness of the instrument can only be assessed following a longitudinal study (internal responsiveness) or in relation to an intervention or change (external responsiveness) [31].

We also recognize that, because we have developed the instrument in one country and in a Swedish-speaking population, it needs to be translated and validated in other languages in order to facilitate the greatest usage. The English translation is ready for validation and we would also welcome other translations, which would allow for pooling of data over larger and more diverse groups.

## Conclusion

There is a general need for enhanced efforts in the field of HrQoL research in all rare diseases. Overall, there is a need for more longitudinal follow-up studies, where health-related quality of life can be addressed. The NARQoL-21 child-centred, self-report measure, which has good discrimination and convergent validity, and excellent internal and external reliability, will prove to provide precise and stable measurement and be a useful addition to the instruments that can be used to evaluate the effects of narcolepsy in a child and adolescent population in longitudinal follow-up. Its inclusion in clinical data registries would be an important step forward to a better understanding of young people’s experience of this health condition.

## Additional files

Additional file 1: Focus Group Summary. (PDF 185 kb)

Additional file 2: NARQoL-21 English–Swedish translation. (PDF 20 kb)

Additional file 3: Table 9. NARQoL-21 cut-off scores for differentiating between optimal and suboptimal HrQoL. (PDF 229 kb)

## Abbreviations

ADHD: Attention deficit hyperactivity disorder;
AUC: Area under the curve
CI: Confidence interval
HrQoL: Health-related quality of life;
ICC: Intraclass Correlation Coefficient
KIDSCREEN: A set of generic quality-of-life instruments for children
KIDSCREEN-10: the KIDSCREEN Mental Health Index for children
NARQoL-21: Narcolepsy quality-of-life instrument with 21 questions;
ROC: Receiver operating characteristic
SD: Standard deviation
