# Supplementary material for: The development of a health-related quality-of-life instrument for young people with narcolepsy: NARQoL-21
Source: Health Qual Life Outcomes. 2017 Jul 4;15:135. doi: 10.1186/s12955-017-0707-8 (PMC5496267; doi:10.1186/s12955-017-0707-8)
Supplement: Supplementary file 1 — Focus Group Summary. (PDF 185 kb) [file 12955_2017_707_MOESM1_ESM.pdf]

Additional file 1. Focus group summary

A focus group study of themes related to the quality of life of adolescents with narcolepsy

John E. Chaplin[1], Attila Szakács[1,2], Tove Hallböök[1], Niklas Darin[1]

[1]Department of Pediatrics, Institute of Clinical Sciences, Sahlgrenska Academy at University of Gothenburg, Gothenburg, Sweden; [2]Department of Pediatrics, Halland Hospital, Halmstad, Sweden.

Corresponding author: John Eric Chaplin Email: john.chaplin@gu.se

Abstract

Background: In order to develop a narcolepsy-specific questionnaire for young people, a series of focus groups were conducted. The objective was to elicit issues related to experiences affecting health-related quality of life (HrQoL) which could be developed into a questionnaire for this population.

Methods: A standardized, 90-minute focus group methodology developed for the DISABKIDS QoL project was used, including a semi-structured interview schedule with topic cards which were sorted for importance. Discussions were digitally recorded and transcribed. A thematic analysis of the item pool was conducted by two of the authors independently generating initial coding of statements and themes. Themes were refined and reviewed by a patient panel of three members of the Swedish Narcolepsy Association and a pediatric neurologist to ensure that all relevant topics were covered. Cognitive debriefings with ten young people further refined the coding and themes.

Results: Twenty young people with narcolepsy (age range 8–18; mean: 13.5 years) participated in four age-defined (8–13 and 15–17 years) mixed gender focus groups during the spring of 2012. Narcolepsy onset was between one and two years prior to participation. The initial thematic analysis produced seven themes with 135 items related to the concept of their HrQoL. These themes were: Emotional support, School performance, Social image, Concern about the future, Being limited by the condition, Personal energy, and Disturbed sleep. Following the cognitive debriefing, a questionnaire of 40 items was available for pilot testing.

Conclusion: The young people in the focus groups found it easy to identify concerns and difficulties in managing everyday life with narcolepsy. They discussed how these issues could potentially lead to limitations in their future lives, expressing feelings about the possibility of missing out on what could be called a ‘good life’. This is not an entirely unique concept in the measurement of HrQoL research, the concepts of *belonging, being* and *becoming* have been discussed in the literature before; however, concerns about the future are often not included as domains in QoL measurement. The themes are illustrated with examples, and the importance of future HrQoL is discussed.

Background

In order to develop a narcolepsy-specific questionnaire for young people, a series of focus groups were conducted. The objective of these was to elicit issues related to their experience of their health-related quality of life (HrQoL) which could be developed into a questionnaire for use within this population. In the development of modern self-assessment questionnaires, the involvement of the individuals in the target populations is considered to be an essential part of the development of a patient-reported outcome measure [1, 2]. HrQoL is viewed as a latent concept and a subjective experience of the patient [3]; therefore, the patients must be involved in the development of the questionnaire [4].

Focus groups are an excellent method for gaining insight into a range of issues related to young people's perspectives on their health and welfare [5]. Focus groups often generate a broad discussion of the issues that can lead to a better understanding of the concepts and perceptions of the patient group, and they generate a large number of potential items for HrQoL questionnaires [6]. It is important to identify as many items related to the impact of the condition as possible in order to improve content validity, responsiveness and reduce random error. However, this must also be balanced with consideration for respondent burden, which is why some method of item reduction is also necessary following the focus group approach.

Child focus groups tend to be different to adult groups because the moderator must consider what questions are appropriate to that age level and must create an atmosphere of acceptance [7]. It is also considered helpful to incorporate activities for the children to do, such as a card sorting exercise or writing draft questions [8]. In this research we also followed the recommendations of the RESPECT project by including a patient panel to further analyze the themes emerging from the focus group data and to increase the participation of the patient group in the development of the questionnaire [9]. In this paper we describe the HrQoL themes generated by the narcolepsy focus groups and describe the item reduction methods used.

Methods

In order to identify relevant questions for measurement of HrQoL in children and adolescents with narcolepsy, a focus group procedure was followed according to a standardized approach developed by DISABKIDS which includes a semi-structured interview guide for the moderators. The method requires two moderators, one who acts as administrator/technician and the other who facilitates the discussion. The goal of the group is not to reach consensus or to discover a single solution but to elicit a full range of feelings or thoughts about a subject. The facilitator seeks to provide a comfortable place for conversation where all views are respected, and tries to avoid pressure on participants to come to agreement. The moderator guide written for the narcolepsy study suggests initial questions aimed at generating general discussion about how narcolepsy could affect the life of a young person. These initial questions were then followed by probes that were sequenced so that they focused on more specific information about experiences recounted by individuals in the group that may stimulate further discussion [6, 8]. The progression of questions and following spontaneous lines of inquiry enabled the facilitator to generate a lively group participation using a multi-stage method converging on specific QoL issues related specifically to narcolepsy. Group membership was based on age. Gender was not considered an important factor in sharing of insights in this population.

The focus group interviews were carried out at the Queen Silvia Children's Hospital, Gothenburg, and at the Ågrenska, Gothenburg, which is the national competence center for rare disorders. Participants were drawn from both a hospital-based population and from week-long awareness courses run by the Ågrenska for young people and adults with disabilities and their families. During the data collection period, two courses were run and focus groups were arranged with their participants. All participants spoke Swedish as their first or second language. The sessions were held over approximately 90 minutes and were divided into two phases. First, the participants were asked a series of semi-structured questions designed to facilitate discussion about their HrQoL issues (see panel 1). Second, topic cards were written and sorted into different piles of importance and then discussed.

Panel 1. Example questions used in the focus groups

Semi-structured questions:
What do you like about your life?
What do you like best about your life?
What makes you happy?
What bothers you most in your life?
What kind of things keep you healthy? (probe for coping activities)

More specific questions:
Tell me about your narcolepsy.
How does narcolepsy affect you at school / at home?
What bothers you most about having narcolepsy? Are there advantages?
Do you know what other kids / young people think of you having narcolepsy?

Four focus groups were conducted during the spring of 2012 with 20 young people with narcolepsy, aged 8–18 years of age, identified in the healthcare regions covering the west of Sweden. The onset of narcolepsy was before 18 years of age and between 1st January, 2000 and 31st December, 2010. Two of the focus groups (with six and four participants, respectively) were held with children aged 8–14 years and two were held with adolescents aged 15–18 years (seven and three participants, respectively). All focus group discussions were digitally recorded and transcribed. Two additional focus groups (with five participants in each) were conducted with ten parents of these patients.

The focus group process was followed by cognitive debriefing with one of the groups and panel discussions to clarify patients' interpretation of the questions in order to refine the questions and ensure that all relevant topics were covered [10]. In order to avoid omitting items that are very important to a substantial number of patients in the final reduced item list, the patient panel was asked to assess the items for those with the highest relevance. Applying the "clinical impact" method described by Guyatt (1986) [11] and by Juniper and colleagues (1997)[12], the most relevant items were then identified. This method is consistent with the definition that quality of life "represents the functional effect of an illness and its consequent therapy upon a patient, as perceived by the patient" [13].

<u>Content analysis</u>
The procedure followed a content analysis and thematic analysis approach outlined by Braun and Clarke (2006) [14]. A categorization derived directly from the text data of the manifest content of the item pool was conducted by two independent researchers (JC & SH) using multiple coding in order to identify and report patterns within the data. The researchers

familiarized themselves with the data via the audio recording and field notes. These two researchers independently generated 15 and 18 initial codes and then reviewed them together. In line with the ambition of using multiple data interpretations to increase the validity of the investigation, a respondent validation procedure was also applied [15]. Following the initial categorization, the items were further evaluated by a patient panel of three people (one woman) who were members of the Swedish Narcolepsy Association. The final thematic analysis of the item pool was based on group discussion with the patient panel. The patient panel met on two occasions to discuss the relevance of the items and to clarify question wording with the consideration that the eventual questionnaire was targeted at adolescents. The themes were then reviewed by a neurologist (AS). It was noted, at this stage, that there were questions related to physical functioning that were missing. However, since physical functioning had not been raised by the patient panel as an important issue, it was decided that the inclusion of an additional theme would not comply with the patient-driven design approach that we had adopted. It was also felt that this issue could be covered by another instrument if necessary. Previous HrQoL studies have found no difference in physical function between narcolepsy patients and the general population in young people [16] or in adults [17].

Results

The initial analysis of the transcribed statements produced a set of seven themes with 135 items of importance to the young people's concept of their QoL with narcolepsy. Issues to do with sleep and physical problems were considered to be better covered by existing validated questionnaires and were therefore dropped from further analysis. The remaining 114 statements were included in the patient panel group analysis which produced seven themes: Emotional support, School performance, Social image, Concern about the future, Being limited by the condition, Personal energy, and Disturbed sleep. The parent groups also produced seven themes with 109 statements: Health and well-being, Independence, Friends, School, Leisure time, Family, and Happiness/contentment with life. At the end of this stage a subset of 40 items – those with the greatest relevance to the conceptual model developed by the focus groups and with the most potential to give an understanding of the wider impact – was used in a pilot questionnaire. The cognitive debriefing exercise indicated that the questions were acceptable, after some minor re-wording suggested by a few of the participants. The participants demonstrated appropriate understanding of the questions in line with expectations. Neither the cognitive debriefing participants nor the patient panel considered any of the questions to be too difficult for the targeted age range.

<u>Differences between the patient and parent focus groups.</u>

As with the patients' focus groups, the analysis of the parent focus groups also produced seven themes, but these tended to focus on the need for medical intervention and were less concerned with the effects of narcolepsy on the child's social life and life at school. This avenue of investigation was therefore not included further in this analysis.

<u>Theme structure from the patient focus groups</u>

1. **School performance**. The problems with school were mentioned by all ages in all focus groups. There were two issues, the first of which was the problem of concentration. It was difficult to stay focused on the task in lessons and this led to poor relationships with teachers ("*the teacher thinks I am lazy*") and feelings of failure ("*I won't get good grades at school*"). The second issue was that of being made fun of in

the classroom and being bullied. Several of the respondents stated that they did not get on particularly well with their fellow pupils and that they did not participate in school activities or sports ("*I don't want to get to know other children in the school*").

2. **Personal energy**. A second theme was that of energy. This was picked up in several descriptions of different activities but it was repeated enough to be seen as a separate theme. The respondents said that they did not have the energy to do things, they were too tired to do sport, not interested in hobbies, tended to withdraw during rest periods at school and could not manage any more than they were already doing ("*I don't really want to do anything*").
3. **Social image of themselves.** In this theme there were two categories: friends and family.
    a. Friends. This was more of an issue for the older children. The younger children talked less about the need for friends and seemed quite content to have the support of their families. For the older children it was a question of not being part of the group ("*I just can't keep up*") and they had difficulty keeping friends because they did not participate in the things that their peers did. A consequence of this was feeling lonely. Again, this was mostly expressed by the older children ("*I would like more friends*"). It was not always that the respondent was rejected, although this issue was raised ("*I feel that I am constantly an object of fun*"), but also that they held themselves back from making friends due to expectations that it would go badly or that they could not offer the same type of friendship that others could offer ("*It is always a problem if I don't do what the others do*").
    b. Family. A theme which had mixed positive and negative issues was that of the relationship with the family. The younger children saw the family as a very great strength. They felt safe only in the family and wanted to be at home most of the time ("*my parents take good care of me, they will always help me*"). However, there were also statements from the older children that they had problems at home ("*I feel I don't get treated fairly at home, I get accused of forgetting things*"). There were also frequent references to not participating in family activities, which could lead to conflict.

4. **Concern about the future.** Considerations about the future emerged from the older children. When talking about school and the expectation of poor grades, the question of what would happen when they left school would often emerge. None of the respondents felt that the future was going to be easy ("*Life as an adult is going to be difficult*") or that they would be successful in their future careers ("*I don't think I will get a good job*") or personal relationships ("*I don't think I will be able to get a girlfriend*"). The feeling was that things would carry on in the same way as they had done at school ("*In my experience you can't avoid being bullied if you have narcolepsy, regardless of whatever job you get.*", "*I'm never taken seriously*").

5. Being limited by the condition. The lack of friends and not making the most of their leisure time meant that several respondents said that they felt they were missing out on life ("*I can't just be spontaneous; it means I miss out on things*"). They also felt that they had to act more responsibly than others of their age because they could not be sure that they would be able to manage a situation or manage to get home without a problem. The older respondents said that they tended to avoid all outings and events because it was just not worth the risk of falling asleep in a strange place ("*I stay at home during the weekends*"). Several said that they did not go to parties, but there were also indications that some of the respondents had established routines which

were accepted by their peers; for example, they would go to a party for the early part of the evening and it was accepted that they left early. They had learnt to manage their situation and had friends who supported them. Those that did not have friends had more problems ("*I don't have anyone to hang out with*"), because it is necessary to have someone to look after you if you fall asleep ("*I really need someone with me all the time*"). Therefore, many of the participants stayed within the family, where there were always people around. This sense of limitation was elevated to an expression of worry about the consequences that this would have for the future.

6. **Emotional support.** A number of issues were raised by both age groups concerning emotional reactions to their situations. Some said they were depressed or anxious all the time or that they felt unsafe and on their guard ("*I feel unsafe*"). This was especially true when taking a bus or tram on their own ("*I must be on my guard all the time*"). The reasons for these emotional reactions was that they felt they could not rely on themselves to stay awake, but also that they experienced nightmares and had disturbing hallucinations. There was also anxiety about getting bullied at school and having to plan for how to avoid this. Several of the older children referred to problems concerning cognitive abilities. These were not mentioned by all participants but they expressed concerns about feeling that they were getting less intelligent ("*I think I get less smart over time*"), and memory led to anxiety ("*I have problems remembering faces*"). On the other hand, a major problem mentioned by several was that they were "*getting reminded about things all the time*" and that they had problems concentrating. Finally, there was a feeling of general discontentment that was seldom expressed but was apparent in many of the discussions ("*I don't feel very contented with life*").

   Several of the respondents talked about getting angry easily and suddenly at times ("*Sometimes I just explode*"); this was always referred to as something that happened at home. Many of these incidents happened around food ("*There is usually an argument with my parents at meal times*"). They were often too tired to eat and this led to arguments with their parents ("*I feel that my parents try to force me to eat – when I don't want to*").

7. **Disturbed sleep and physical issues.** Sleep emerged as an issue which the respondents felt reduced their QoL. They had to sleep during the day, could not read without falling asleep, could not watch TV, and it was pointless going to the cinema because they would fall asleep as soon as the lights faded. There were very few physical issues, but some respondents said that it was sometimes a problem that they could not keep their arms or legs still; however, this was not related to QoL.

<u>Item reduction and cognitive debriefing</u>

Duplication and ambiguous items were removed and the remaining items were ranked in order not to miss important issues. The objective was to reduce the number of items by identifying those that were the most important and most likely to detect change (responsiveness). The item frequency in the focus groups was assessed by the number of statements related to each item. This gave a simple and reasonable method to reduce items. After initial coding, seven preliminary themes were suggested. These were further refined and defined with reference to the original data and in cognitive debriefing with 10 young people with narcolepsy (aged 16–18, four girls). Participants completed the pilot version of the questionnaire (40 questions) with a 'thinking aloud' procedure to explain the reason for

responses. A subsequent cognitive debriefing established whether the participants understood the question wording, interpreted the questions as intended, and felt able to use the response scale appropriately. They were also given the opportunity to add any relevant concepts that they thought were missing and suggest rewording of the questions. Different interpretations were discussed, and a further content analysis was conducted by a pediatric neurologist (AS) before agreement on definitions was achieved with example quotes.

Panel discussions, cognitive debriefing, and clinical assessment resulted in the elimination of redundant items with similar meaning and items causing any confusion. The items and themes were assessed by the patient panel, which helped to identify the most relevant items and clarify question wording. The patient panel identified two sets of critical items: those related to current HrQoL and those related to the future. A final list of 27 HrQoL questions formed the core questions of the pilot NarQoL. In addition, nine items relating to concerns about the future were identified and included in further analysis as mediators of HrQoL.

Discussion

The focus groups highlighted the additional strains on the participants' lives due to having narcolepsy. A negative impact was described principally in school where relationships with the teachers and the other pupils were mentioned. These relationships are participially important. It has been shown that a decline in the teacher-student relationship can lead to increases in depression among the children [18] and it has been shown that the stigma has been shown to be an important determinant of lower quality-of-life in young people with narcolepsy [19]. Our focus findings would support the view that being accepted for the person that they are is a major strain for young people with narcolepsy.

The focus group participants also reported that narcolepsy made them miss-out on activities. Adolescence can be a frustrating time for anyone but if you are also someone with a disability then there is the added difficulty of becoming independent which challenges the parental support network that is doubly important for someone with a disability. Adolescence is characterized by acts of independence, which include going to school without your parents and independently using public transport [20]. These are exactly the sort of activities that are difficult for someone with narcolepsy.

The participants mentioned that they would like more friends and they talked about the necessity of having understanding friends in order to feel that they belong to a group. Regular contact with friends has been recognized as an important determinant of positive physical and psychological health [21] and helps to establish social skills, which are useful when becoming an adult [22]. If young people with narcolepsy are being isolated from their peers then, it will be advisable to provide opportunities to form friendships. Additional research into the quality of peer relationship in this population should be undertaken.

Conclusion

The young people in the focus groups found it easy to identify concerns and difficulties in managing everyday life with narcolepsy. They discussed how these issues could potentially lead to limitations in their future lives, expressing concerns about the possibility of missing out on what could be called a 'good life'. This is not an entirely unique concept in the measurement of HrQoL research, as the concepts of *belonging*, *being* and *becoming* have been discussed in the literature before [23]. However, concerns about the future are rarely included as domains in HrQoL measurement.

Declarations

Ethics approval and consent to participate

Ethical approval for the study procedures was given by the Regional Board of Medical Ethics at the University of Gothenburg (ref: 246-11). All patients, control subjects, and their parents received a letter with information about the study and all consented to participation. All procedures were in accordance with the Helsinki Declaration as revised in 2013.

Consent for publication

Not applicable.

Availability of data and supporting materials

The datasets generated and analyzed during the current study are not publicly available for confidentiality reasons but are available from the corresponding author upon reasonable request.

Competing interests

The authors declare that they have no competing interests.

Funding

Funding was obtained from Health Region Halland, Sweden (FF-2012-009). The funding body had no role in the design of the study or collection, analysis, and interpretation of data, and did not contribute to the writing of the manuscript.

Authors' contributions

JEC carried out the focus group interviews, data analysis and drafted the manuscript. AS conducted the content analysis. AS and JEC jointly drafted the manuscript. TH and ND participated in the design and coordination of the study and helped to draft the manuscript. All authors read and approved the final manuscript.

Acknowledgements

The authors would like to thank the focus group participants who shared their experiences and thoughts with us. Special thanks to Sarah Hitz from Dept. of Health Science, Faculty of Life Sciences, Hamburg University of Applied Sciences, Hamburg, Germany who assisted with the content analysis. We are grateful to Ågrenska National Competence Centre for Rare Diseases for allowing us to participate in their narcolepsy family weeks and talk to the participants. We would also like to express our deepest thanks to all the participants and members of the patient panel. We thank CMC Scientific English (*www.scientificenglish.se*) for help with proofreading this manuscript.

References

1. European Medical Agency: **Reflection paper on the regulatory guidance for the use of health-related quality of life (HRQL) measures in the evaluation of medicinal products.** (European Medical Agency London: Committee for Medicinal Products for Human use; 2005.
2. Food and Drug Administration: **Guidance for Industry: Patient-Reported Outcome Measures.** (Food and Drug Administration USDoHaHS Silver Spring, MD: Office of Communications; 2009.
3. Ravens-Sieberer U, Devine J, Bevans K, Riley AW, Moon J, Salsman JM, Forrest CB: **Subjective well-being measures for children were developed within the PROMIS project: presentation of first results.** *J Clin Epidemiol* 2014, **67:**207-218.
4. O'Cathain A, Murphy E, Nicholl J: **Why, and how, mixed methods research is undertaken in health services research in England: a mixed methods study.** *BMC Health Serv Res* 2007, **7:**85.
5. Peterson-Sweeney K: **The use of focus groups in pediatric and adolescent research.** *J Pediatr Health Care* 2005, **19:**104-110.
6. Baars R, Chaplin J, Koopman HM and the DISABKIDS group: **A child focus group methodology: experiences from the European DISABKIDS project.** In *Paediatric Health Related Quality of Life: a European Perspective Instrument development, validation, and use in clinical practice.* Leiden: Leiden University Open Access
7. Horowitz JA, Vessey JA, Carlson KL, Bradley JF, Montoya C, McCullough B: **Conducting school-based focus groups: lessons learned from the CATS project.** *J Pediatr Nurs* 2003, **18:**321-331.
8. Chaplin JE: **DISABKIDS Focus Group Manual.** vol. April 2017, 4 edition. pp. Outline of the focus group procedure used in DISABKIDS and subsequent projects. Gothenburg, Sweden: University of Gothenburg; 2015:Outline of the focus group procedure used in DISABKIDS and subsequent projects.
9. Chaplin JE, Crawley FP, Sanna L, Ceci A, Neubauer D, Giaquinto C, Chaplin C, Bullinger M: *RESPECT Patient Needs* Lengerich: Pabst Science; 2012.
10. Heary CM, Hennessy E: **The use of focus group interviews in pediatric health care research.** *J Pediatr Psychol* 2002, **27:**47-57.
11. Guyatt GH, Bombardier C, Tugwell PX: **Measuring disease-specific quality of life in clinical trials.** *CMAJ* 1986, **134:**889-895.
12. Juniper EF, Guyatt GH, Streiner DL, King DR: **Clinical impact versus factor analysis for quality of life questionnaire construction.** *J Clin Epidemiol* 1997, **50:**233-238.
13. Schipper H Clinch J, Powell V. : **Definitions and conceptual issues.** In *Quality of life assessments in clinical trials.* Edited by Spilker B. New York: Raven Press; 1990: 11–35
14. Braun B: **Phonetics and phonology of thematic contrast in German.** *Lang Speech* 2006, **49:**451-493.
15. Barbour RS: **Checklists for improving rigour in qualitative research: a case of the tail wagging the dog?** *BMJ* 2001, **322:**1115-1117.
16. Rocca FL, Finotti E, Pizza F, Ingravallo F, Gatta M, Bruni O, Plazzi G: **Psychosocial Profile and Quality of Life in Children With Type 1 Narcolepsy: A Case-Control Study.** *Sleep* 2016, **39:**1389-1398.
17. David A, Constantino F, dos Santos JM, Paiva T: **Health-related quality of life in Portuguese patients with narcolepsy.** *Sleep Med* 2012, **13:**273-277.

18. Reddy R, Rhodes JE, Mulhall P: **The influence of teacher support on student adjustment in the middle school years: a latent growth curve study.** *Dev Psychopathol* 2003, **15:**119-138.
19. Kapella MC, Berger BE, Vern BA, Vispute S, Prasad B, Carley DW: **Health-related stigma as a determinant of functioning in young adults with narcolepsy.** *PLoS One* 2015, **10:**e0122478.
20. Shepperdson B: **Negotiating adolescence.** In *Transitional change in the lives of people with intellectual disability* Edited by May D. London: Jessica Kingsley; 2000: pp. 55-75
21. Marmot M, Wilkinson R: *Social Determinants of Health.* Oxford: Oxford University Press; 1999.
22. Buhrmester D: **Intimacy of friendship, interpersonal competence, and adjustment during preadolescence and adolescence.** *Child Dev* 1990, **61:**1101-1111.
23. Renwick R, Brown I: **Being, belonging, becoming: The Centre for Health Promotion model of quality of life. .** In *Quality of life in healt h promotion and rehabilitation: Conceptual approaches, issues, and applications* Edited by Renwick RB, I., & M. Nagler. Thousand Oaks, CA: Sage; 1996: 88
