# Supplementary material for: The development of a health-related quality-of-life instrument for young people with narcolepsy: NARQoL-21
Source: Health Qual Life Outcomes. 2017 Jul 4;15:135. doi: 10.1186/s12955-017-0707-8 (PMC5496267; doi:10.1186/s12955-017-0707-8)
Supplement: Supplementary file 2 — NARQoL-21 English–Swedish translation. (PDF 20 kb) [file 12955_2017_707_MOESM2_ESM.pdf]

Additional file 2. NARQoL21 English / Swedish versions

	English	Swedish
1	I can keep up in lessons.	Jag hänger med på lektioner.
2	I feel more lazy than others.	Jag känner mig mer lat än andra.
3	I can sit still and concentrate.	Jag kan sitta still och koncentrera mig.
4	I feel sad or low.	Jag känner mig ledsen eller deppig.
5	I see obstacles for me in the future.	Jag ser hinder för mig i framtiden.
6	I think I will get a driving license.	Jag tror jag kommer att få körkort.
7	I stay awake when I read	Jag håller mig vaken när jag läser
8	Everything will be fine for me in the future.	Allt kommer att gå bra för mig i framtiden
9	I get angry very easily.	Jag blir väldigt lätt arg.
10	I give up easily.	Jag ger lätt upp.
11	I have problems to be awake all the time in school.	Jag har problem med att vara vaken hela tiden i skolan.
12	I feel lonely.	Jag känner mig ensam.
13	I find it easy to get along with others.	Jag har lätt att komma överens med andra.
14	I will not travel as much as others.	Jag kommer inte att resa så mycket som andra.
15	I get anxious.	Jag får ångest.
16	There will be good opportunities for me.	Det kommer att finnas bra möjligheter för mig.
17	I feel sure of myself.	Jag känner mig säker på mig själv.
18	I stay awake when I watch TV / movies.	Jag håller mig vaken när jag tittar på tv/bio.
19	Everything is going to be worse when I leave school.	Allt kommer bli sämre när jag slutar skolan
20	I am sulky or irritated.	Jag är sur eller irriterad.
21	I stay awake on local public transport	Jag håller mig vaken på lokaltrafik.

An English version of the NARQoL-21 was translated using several steps: an initial forward translation (from Swedish to English) by two independent translators with a background in linguistics and quality of life research; a back translation (from English to Swedish), and finally a consensual version to check against the original NARQoL by all three translators and the originator of the instrument. Discrepancies were discussed and agreement achieved.
