# Supplementary material for: The development of a health-related quality-of-life instrument for young people with narcolepsy: NARQoL-21
Source: Health Qual Life Outcomes. 2017 Jul 4;15:135. doi: 10.1186/s12955-017-0707-8 (PMC5496267; doi:10.1186/s12955-017-0707-8)
Supplement: Supplementary file 3 — Table 9. NARQoL-21 cut-off scores for differentiating between optimal and suboptimal HrQoL. (PDF 229 kb) [file 12955_2017_707_MOESM3_ESM.pdf]

Additional file 3. Table 9.

NARQoL-21 cut-off scores differentiating between optimal & suboptimal HrQoL

NARQoL-21 score	Sensitivity %	1 – Specificity %
19	0	0
23	5	0
27	5	1
28	11	1
31	16	1
34	26	1
35	32	1
36	53	1
38	63	2
39	63	3
41	74	3
42	79	3
43	79	4
45	79	5
**47**	**84**	**8**
48	84	9
49	84	10
51	84	12
53	84	12
54	84	14
